# Oleandrin induces DNA damage responses in cancer cells by suppressing the expression of Rad51

**DOI:** 10.18632/oncotarget.10726

**Published:** 2016-07-20

**Authors:** Zhengqiang Bao, Baoping Tian, Xiaohui Wang, Hanrong Feng, Ye Liang, Zhihua Chen, Wen Li, Huahao Shen, Songmin Ying

**Affiliations:** ^1^ Department of Respiratory and Critical Care Medicine, Second Affiliated Hospital, Institute of Respiratory Diseases, Zhejiang University School of Medicine, Hangzhou 310009, China; ^2^ Department of Pharmacology, Zhejiang University School of Medicine, Hangzhou 310058, China; ^3^ State Key Laboratory of Respiratory Diseases, Guangzhou, Guangdong 510120, China

**Keywords:** oleandrin, DNA damage response, DNA replication, apoptosis, single strand break repair

## Abstract

Oleandrin is a monomeric compound extracted from leaves and seeds of Nerium oleander. It had been reported that oleandrin could effectively inhibit the growth of human cancer cells. However, the specific mechanisms of the oleandrin-induced anti-tumor effects remain largely unclear. Genomic instability is one of the main features of cancer cells, it can be the combined effect of DNA damage and tumour-specific DNA repair defects. DNA damage plays important roles during tumorigenesis. In fact, most of the current chemotherapy agents were designed to kill cancer cells by inducing DNA damage. In this study, we found that oleandrin was effective to induce apoptosis in cancer cells, and cause rapid DNA damage response, represented by nuclear RPA (Replication Protein A, a single strand DNA binding protein) and γH2AX(a marker for DNA double strand breaks) foci formation. Interestingly, expression of RAD51, a key protein involved in homologous recombination (HR), was suppressed while XRCC1 was up-regulated in oleandrin treated cancer cells. These results suggested that XRCC1 may play a predominant role in repairing oleandrin-induced DNA damage. Collectively, oleandrin may be a potential anti-tumor agent by suppressing the expression of Rad51.

## INTRODUCTION

Oleandrin is a monomeric compound extracted from leaves and seeds of Nerium oleander, with a molecular weight of 576.73 and molecular formula of C_32_H_48_O_9_. As a kind of cardiac glycosides, it was well known for its effect in treating congestive heart failure [1]. Not only that, in the past decades, more and more studies have revealed that oleandrin may be a potential anti-tumor agent, as oleandrin can effectively inhibit proliferation of various cancer cells and induce apoptosis [2–5]. Besides that, oleandrin can also enhance the efficiency of radiotherapy [6]. Interestingly, the anti-tumor role of oleandrin seemed to be selective, as oleandrin can kill certain human tumor cells but not murine tumor cells [7, 8]. In a phase I study, oleandrin was used to remedy patients with refractory solid tumors, where oleandrin was found to be well tolerated and only few adverse events were reported [9]. Up to now, various studies on many possible pathways have been made to elucidate the anti-cancer role of oleandrin. Some argues that oleandrin's ability to inhibit cancer cells proliferation were because of the decrease in levels of Na, K-ATPase [10]. Mitochondrial injury caused by the generation of reactive oxygen species (ROS) was also taken into account [11]. Others suggest that activation of caspase-3 by oleandrin may be a cause of tumor cells apoptosis [6]. It had been reported that cancer cells were arrested in G2/M cell cycle by oleandrin [12], suggesting activation of DNA damage checkpoint. However, detailed mechanisms of the anti-tumor role of oleandrin are still not fully understood.

As we know, Genomic instability is one of the main features of cancer cells, it can be the combined effect of DNA damage and tumour-specific DNA repair defects [13], and plays important roles during tumorigenesis. At the present, many chemotherapy agents were designed to target DNA damage repair to induce cancer cell apoptosis. Here, we investigated roles of oleandrin in induction of cancer cell apoptosis as well as its impact on DNA damage response.

## RESULTS

### Oleandrin Induces cell death in multiple cancer cell lines

**Figure 1:**
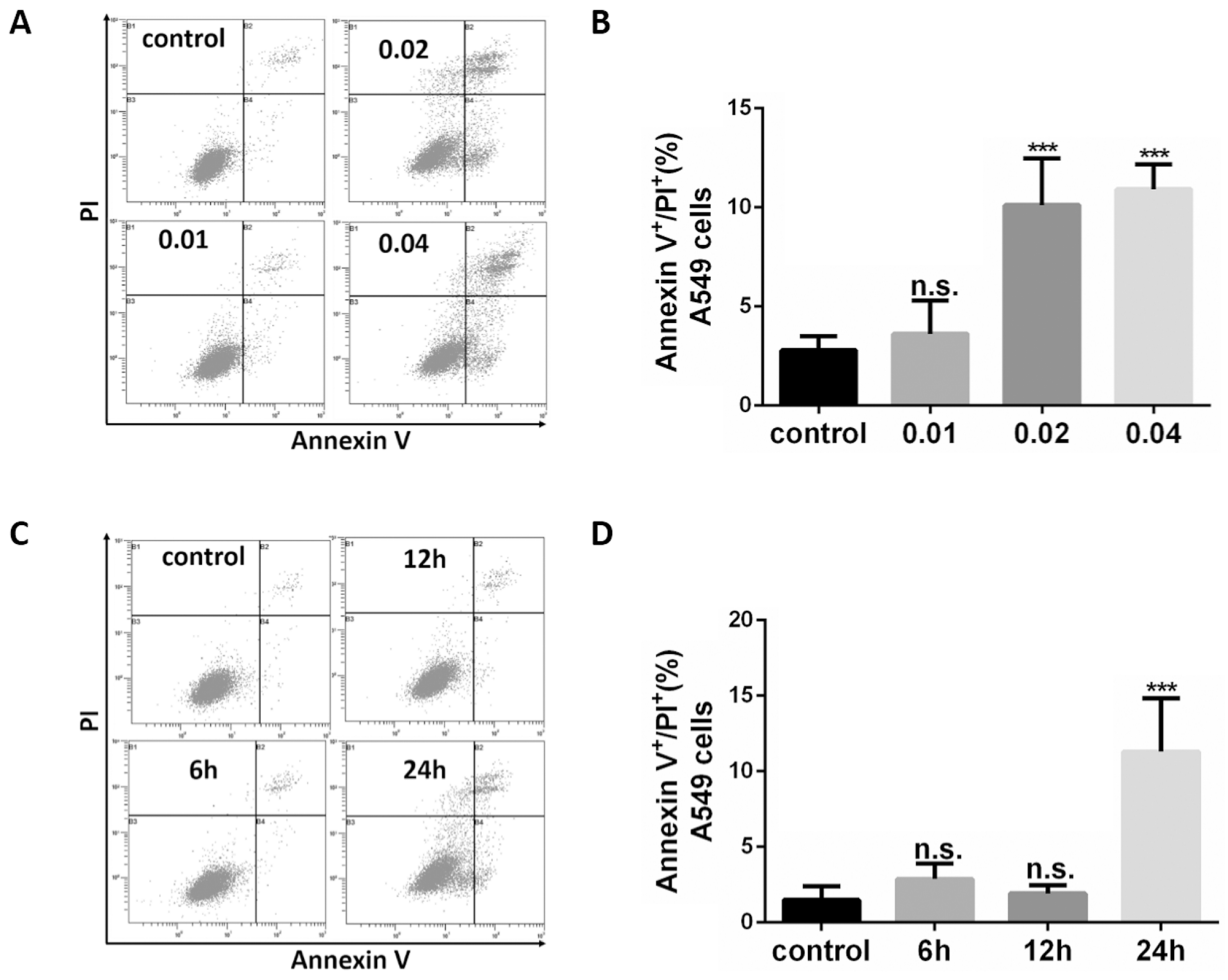
Oleandrin induced apoptosis in A549 cells **A.** Representative FACS profiles of A549 cells treated with different concentrations of oleandrin for 24 hours. **B.** Quantification of Annexin V positive and PI positive A549 cells following treatment of oleandrin at indicated concentrations were shown. **C.** Representative FACS profiles of A549 cells treated with oleandrin (0.02 ug/ml) for different hours (6h, 12h, 24h). **D.** Quantification of Annexin V positive and PI positive A549 cells following treatment of oleandrin for indicated hours were shown. All data represent mean ± SEM (n=3); n.s. not significant, ***P<0.001, compared with the controls.

**Figure 2:**
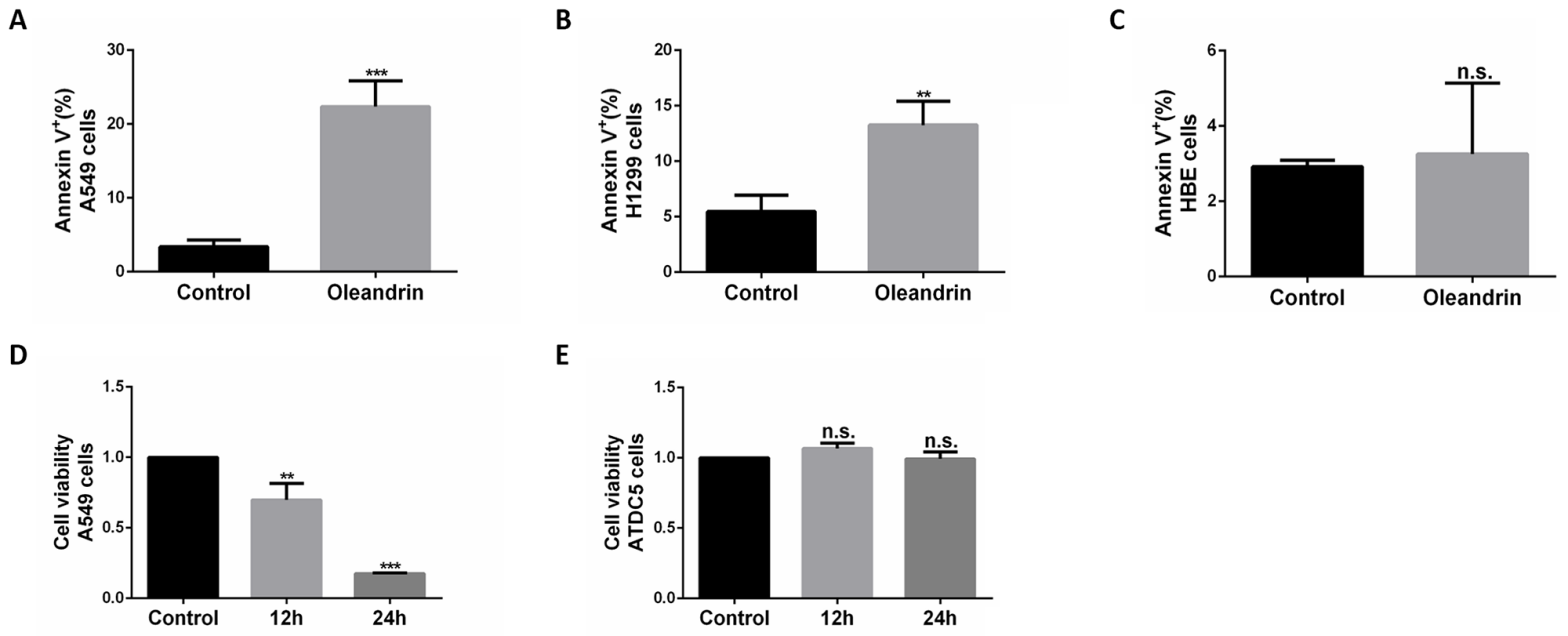
Oleandrin effectively induced cell death in A549 and H1299 cells, but not in HBE and ATDC5 cells **A.** Quantification of cell death in A549 cells treated with oleandrin (0.02ug/ml) for 24 hours, detected by FACS. **B.** Quantification of cell death in H1299 cells treated with oleandrin (0.02ug/ml) for 24 hours, detected by FACS. **C.** Quantification of cell death in HBE cells treated with oleandrin (0.02ug/ml) for 24 hours, detected by FACS. **D, E.** Quantification of cell viability in A549 cells and ATDC5 cells treated with oleandrin (0.02ug/ml) for 24 hours, detected by CCK-8 assay. All data represent mean±SEM(n=3); n.s. not siginificant; **P<0.01; ***P<0.001, compared with controls.

To better understand how oleandrin induces cancer cell apoptosis, A549 cells were treated with oleandrin, followed by detection of apoptosis by flow cytometry (FCM). Increased concentrations of oleandrin (0.01ug/ml, 0.02ug/ml, 0.04ug/ml) were incubated with A549 cells for 24 hours, where as low as 0.02 ug/ml was found to be sufficient in apoptosis induction (Figure 1A, 1B). Compared with the control group, apoptosis of A549 cells with treatment of oleandrin (0.02ug/ml, 0.04ug/ml) groups showed a statistically significant increase. Furthermore, apoptosis was induced by oleandrin (0.02ug/ml) in a time-dependent manner (Figure 1C, 1D). Similar experiments were performed in additional two cell lines, including HBE (a human bronchial epithelial cell line) and H1299 (a human non-small cell lung carcinoma cell line). Interestingly, the two cancer cell lines were more sensitive to oleandrin treatment, while HBE cells showed little toxicity (Figure 2A-2C).

In addition, we also detected the toxicity of oleandrin in A549 cells and ATDC5 cells(a normal cartilage cell line) with the method of CCK-8 assay, found that the cell viability of A549 cells were largely limited, while it did no harmful to ATDC5 cells (Figure 2D, 2E).

### Oleandrin induces DNA damage response in lung cancer cells

**Figure 3:**
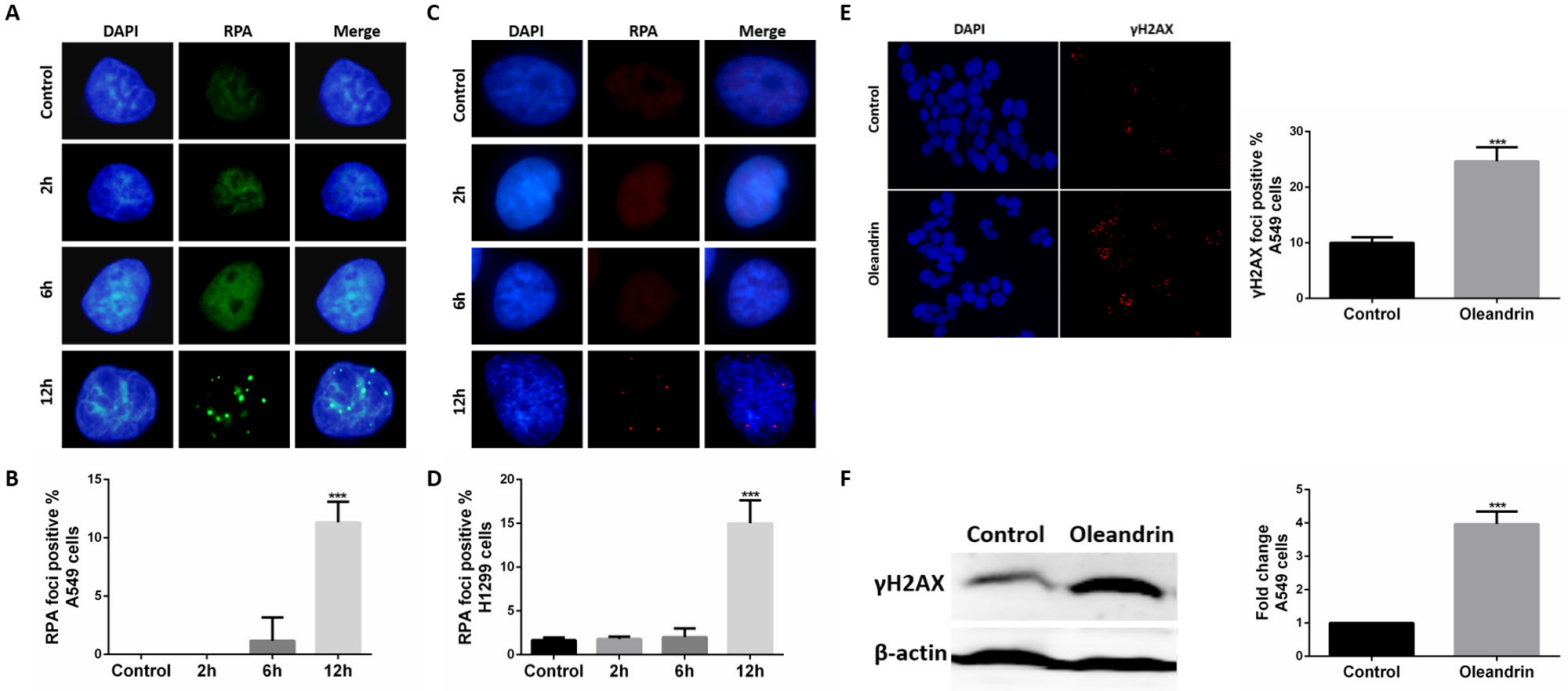
Oleandrin induced RPA and gH2AX foci in lung cancer cells **A.** Immunofluorescence staining of RPA in A549 cells treated with oleandrin (0.02ug/ml) for 2hours, 6hours, 12hours. **B.** Quantification of (A). **C.** Immunofluorescence staining of RPA in H1299 cells treated with oleandrin (0.02ug/ml) for 2hours, 6hours, 12hours. **D.** Quantification of (C). **E.** Immunofluorescence staining of γH2AX in A549 cells treated with oleandrin (0.02ug/ml) for 24hours. **F.** A549 cells treated with oleandrin (0.02ug/ml) for 24hours, γH2AX was analyzed by western blot. All data represent mean±SEM (n=3). ***P<0.001, compared with controls.

RPA is a ubiquitous eukaryotic single-stranded DNA (ssDNA) binding protein that serves to protect every generated ssDNA from degradation [14]. With the method of immunofluorescence, we measured localization of RPA in A549 and H1299 cells following treatment with oleandrin (0.02 ug/ml). It was obvious that oleandrin could significantly increase levels of RPA foci formation at 12 hours (Figure 3A-3D), at which point no significant cell death was yet detected (Figure 1).

To investigate whether oleandrin could induce DNA damage in cancer cells, we measured localization of γH2AX, a marker for DNA double-strand breaks (DSBs), in A549 cells following treatment with oleandrin (0.02 ug/ml) for 24 hours. It was obvious that oleandrin could significantly induce γH2AX foci formation (Figure 3E). To confirm this, we also detected the expression of γH2AX with the method of western blot, and the results (Figure 3F) were consistent with those in immunofluorescence.

### Oleandrin down-regulates expression of HR protein RAD51

**Figure 4:**
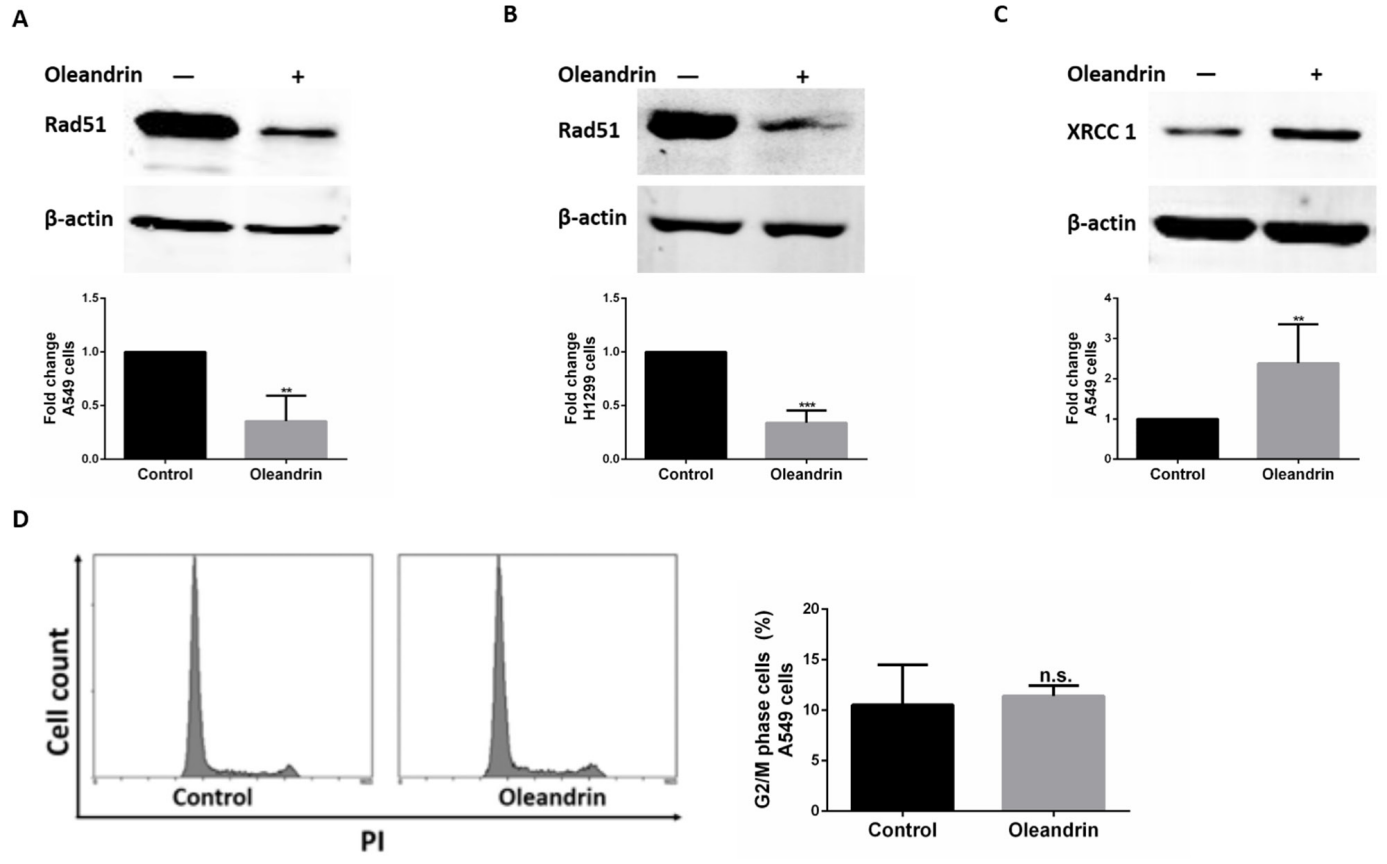
Expression of DNA damage repair proteins in cancer cell lines following treatment with oleandrin **A.** Western blot analysis of RAD51 and Actin in A549 cells. Quantification of RAD51 expression level was performed by densitometric analysis. **B.** Western blot analysis of RAD51 and Actin in H1299 cells. Quantification of RAD51 expression level was performed by densitometric analysis. **C.** Western blot analysis of XRCC1 in A549 cells. Quantification of XRCC1 expression level was performed by densitometric analysis. **D.** Cell cycle of A549 cells treated with oleandrin (0.02ug/ml) for 24 hours. Percentage of cells stalled in G2/M phase were quantified. All data represent mean±SEM (n=3). n.s. not significant; **P<0.01; ***P<0.001, compared with controls.

There are two major DSB repair pathways in higher eukaryotes, HR and non-homologous end joining (NHEJ). And, HR plays an important role in DSB repair. To ensure whether the DNA breaks-induced by oleandrin were repaired by HR pathway. We measured the expression of RAD51 by western blot. In fact, the expression level of RAD51 reduced significantly in A549 and H1299 cells following treatment with oleandrin (Figure 4A-4B). XRCC1-mediated single-strand breaks repair(SSBR) plays a compensatory role to RAD51-mediated HR pathway. We therefore measured levels of XRCC1 to test whether the DSBs induced by oleandrin were repaired through SSBR pathway. Not surprisingly, cells treated with oleandrin showed increased expression levels of XRCC1 protein (Figure 4C). As HR is usually cell cycle dependent. To rule out the possibility of cell cycle arrest-induced down-regulation of RAD51, we measured cell cycle profile of oleandrin-treated cells, and did not detect any significant alteration of G2/M population (Figure 4D).

### XRCC1 is required for cell survival against oleandrin-induced DNA damage

**Figure 5:**
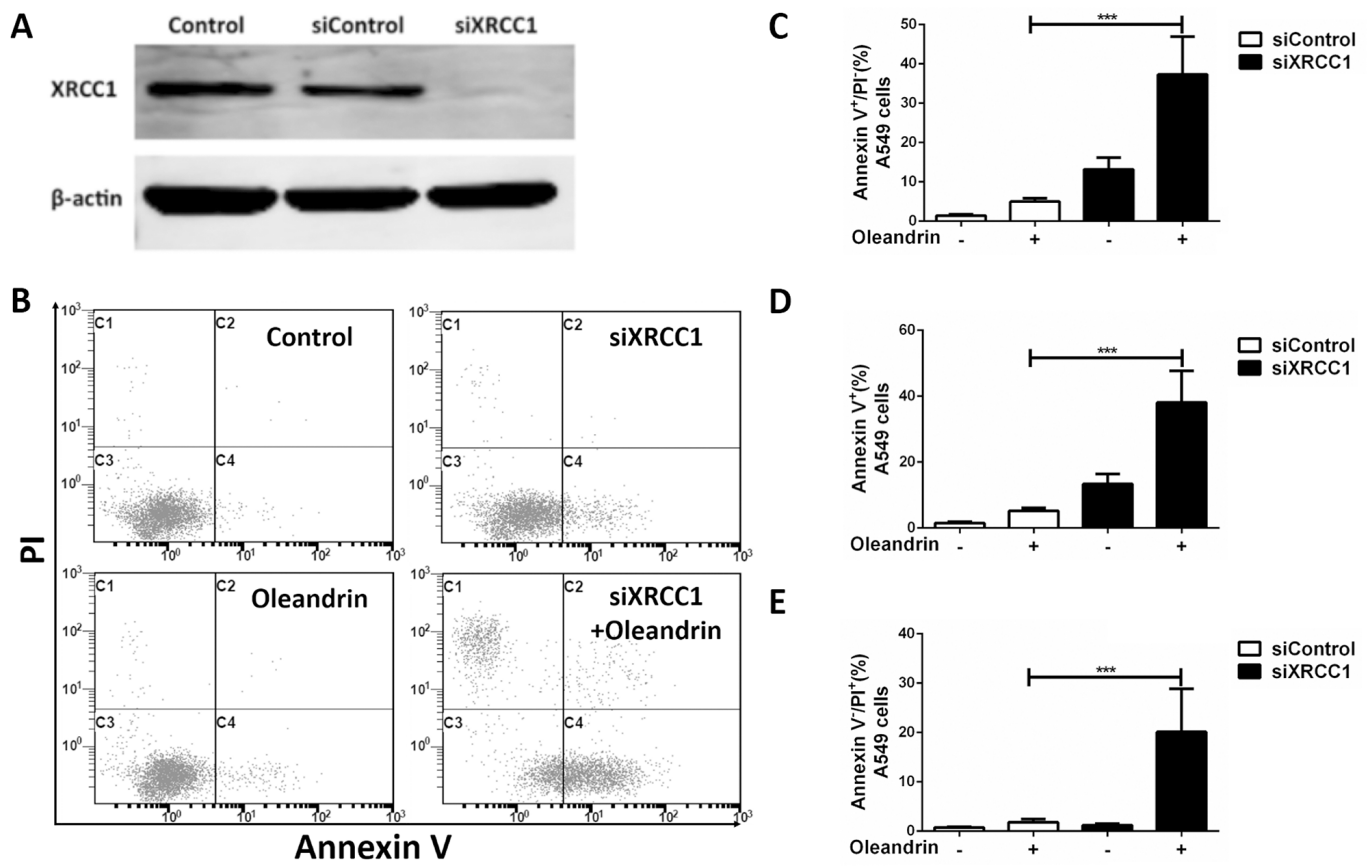
Loss of XRCC1 sensitized A549 cells to oleandrin-induced cell death **A.** Western blot analysis of XRCC1 and Actin in A549 cells following indicated siRNA transfection. **B.** A549 cells were incubated with oleandrin (0.02ug/ml) for 12 hours, 24 hours after the transfection of XRCC1 siRNA. Cell death was detected with FACS analysis. **C, D, E.** Quantification of (B) for indicated values. All data represent mean±SEM (n=3). ***P<0.001.

We have now shown that Rad51 was significantly decreased, while XRCC1 expression level was elevated by oleandrin. In order to explore potential roles of XRCC1 in repair against oleandrin-induced DNA damage, we knocked down XRCC1 by siRNA strategy with satisfactory efficiency (Figure 5A). Interestingly, depletion of XRCC1 was found to significantly sensitize cells to oleandrin-induced cell death (Figure 5B-5E), suggesting that XRCC1 may play an essential role in promoting cell survival following oleandrin treatment.

## DISCUSSION

Oleandrin is a lipid-soluble cardiac glycoside, with a molecular weight of 576.73 and molecular formula of C_32_H_48_O_9_, the main cardiac glycoside in Nerium oleander L. (Apocynaceae). Consistent with previous report [12], oleandrin was able to induce cancer cell death. We found that oleandrin at a low concentration of 0.02ug/ml could significantly induce death of A549 following a 24h treatment, indicating that oleandrin may be a potential effective anti-tumor agent. Interestingly, the anti-tumor role of oleandrin seemed to have some selectivity, as it was more harmful to lung cancer cells than normal bronchial epithelial cells. And in ATDC5 cells, it also presented little toxicity by our results. As we know, it had not been reported at one time. We thought the possible reason to explain the different roles of oleandrin between cancer cells and normal cells may be DNA damage. DNA damage is one reason of tumorigenesis, and most of tumors have genetic DNA defects, while normal cells usually does not have these problems. In fact, many strategies have been made to treat tumor target DNA damage, take Olaparib(a DNA damage repair inhibitor) for example, it was approved by FDA for its benefit in BRCA deficiency cancers [15], Olaparib was particular effective to HR pathway deficiency tumors. Our results also presented that oleandrin could reduce RAD51 expression and may inhibit HR pathway, this may increase the sensitivity of cancer cells to oleandrin. However, we can't deny that oleandrin is a kind of poison agent, and the selectivity of its toxicity was bound. And the detailed mechanisms of this were still unknown and further study need to be done to elucidate it.

In our study, RPA foci was significantly increased by oleandrin. As a major single-stranded DNA binding protein, RPA plays crucial roles in almost all aspects of eukaryotic DNA metabolism include DNA replication, DNA repair, recombination, cell cycle and DNA damage checkpoints [16]. It is abundant in eukaryotic cells, and can bind to single-stranded DNA with sub-nanomolar affinity [17]. So, any single-stranded region formed in DNA will be immediately bound by RPA. The essential role of RPA is to preserve genome stability [18]. It contributed to the early stages of DNA damage signaling cascade and DNA repair [16, 19]. So the increased level of RPA foci indicated increased levels of single-stranded DNA region in genomic DNA. If these single-stranded DNA were not repaired in time, they are likely to be transferred into DNA double-strand break, and the expression of γH2AX may be detected. According to our results, oleandrin induce the increased foci of RPA at an early time of 12 hours, and at this time little toxicity was presented until 24 hours. All this may reflect the potential role of RPA in the death of tumor cells, and increased risk of genetic instability induced by oleandrin.

γH2AX is a sensitive marker of DSBs [20, 21]. DSBs are considered to be one of the most severe forms of DNA damage to a cell, as DSBs can lead to genomic instability and cell apoptosis if not repaired in proper way [22]. When a DSB happened, the histone 2A (H2A)X was rapidly phosphorylated at ser139 by PI3K- like kinase, including ATM (ataxia telangiectasia mutated)/ATR (ataxia telangiectasia and Rad3-related) and DNA-dependent protein kinase [23, 24]. And the phosphorylated variant H2AX is referred as γH2AX. It is also an important player in preserving genome integrity, it attaches to chromatin near DSBs and induces the repair of damaged DNA [25]. Then, it recruits DNA damage repair proteins to the sites of DSBs and initiates the DNA damage signal transduction, and determines whether the damaged DNA will be repaired. When the DNA damage was repaired, γH2AX would be dephosphorylated to H2AX, and enter normal cell cycle [26, 27]. Usually, the presence of γH2AX indicating that some of the DNA damage remains unrepaired. In our study, a lot more γH2AX foci were seen in the oleandrin-induced A549 cells than that in the control group, and western blot assay also confirmed this. Taken together, we think oleandrin induces DNA damage in cancer cells. As mentioned above, high level of DNA damage induced by oleandrin may not all be repaired in a proper way, and may contribute to the death of cancer cells.

HR and NHEJ are two major DNA breaks repair pathways in higher eukaryotes. And HR is the most conservative pathway to repair DSBs and usually happens during S and G2 phases of cell cycle, it requires highly homology for the repair of DSBs [28, 29]. While NHEJ, using limited or no homology for end joining, has a minimal role to repair DSBs. Compared to HR pathway, NHEJ is a “quick and dirty” process, but less accurate [30–32]. However, it provides a compensatory way to DSBs repair when the HR pathway was blocked. NHEJ pathway is considered to be a possible way that enables cells to maximize their chances of survival [32]. In this study, to elucidate the mechanism of DNA repair in oleandrin induced anti-tumor effect, we measured expression of DNA damage repair proteins RAD51 and XRCC1 in oleandrin-treated cancer cells.

If the DNA breaks-induced by oleandrin were mainly repaired by HR pathway, an increased level of RAD51 expression might be observed. However, the expression level of RAD51 in oleandrin-treated cancer cells showed a significant reduction. RAD51 is a key protein in HR and plays an important role in cellular proliferation by repairing DNA damage. It had been reported that the inhibitor of RAD51 weakens HR pathway and sensitizes cancer cells to anti-tumor therapy [33]. To rule out the possibility of cell cycle arrest-induced down-regulation of RAD51, we measured cell cycle profile of oleandrin-treated cells, and did not detect any significant alteration of G2/M population. Taken together, the decreased level of RAD51 induced by oleandrin may contribute to the inhibition of HR pathway, and lead to the death of cancer cells.

As we know, XRCC1 plays an important role in single strand break repair, and backup nonhomologous end-joining pathway. XRCC1 mediated SSBR also plays a compensatory role to RAD51-mediated HR pathway. We therefore measured the level of XRCC1 to test whether the DSBs induced by oleandrin were repaired through SSBR pathway. As expected, A549 cells treated with oleandrin showed an increased level of XRCC1 expression. XRCC1 is a critical factor in SSBR, it functions as a molecular scaffold protein and involved in SSBR by interacting with DNA glycosylases, apurinic/apyrimidinic endonulcease (APE1), PARP-1, polynulceotide kinase (PNK), and ligase III [34, 35]. In our study, the increased level of XRCC1 expression indicated the activation of XRCC1 induced DNA repair pathway such as SSBR, and this may be due to the inhibition of HR pathway, and to activate downstream repair machinery in response to oleandrin-induced DNA strand breaks. We also did a further study that A549 cells were more sensitive to oleandrin when XRCC1 was knocked down, suggesting that XRCC1 may berequired for cell survival against oleandrin-induced DNA damage.

In addition, as presented in Fiugre 5E, there is a small part of cancer cells were stained with PI. Annexin V represent the status of early apoptosis; while PI would emerge in late apoptosis or necrosis cells. So we think the death of cancer cells induced by oleandrin mainly through apoptosis, but apoptosis was not the only, necrosis may also existed.

## CONCLUSIONS

In conclusion, oleandrin could effectively induce the death of cancer cells at a very low concentration; the anti-tumor role of oleandrin may be related with DNA damage repair; and oleandrin may be a novel HR inhibitor by suppressing the expression of Rad51.

## MATERIALS AND METHODS

### Cell culture

The lung cancer cell lines A549 and H1299 were grown in DMEM/ high glucose medium (HyClone) with 10% Fetal Bovine Serum at 37 °C under an atmosphere containing 5% CO2. The normal epithelial cell line HBE was grown in RPMI Medium 1640 basic (1x) (Gibco by life technologies) with 10% Fetal Bovine Serum at 37 °C under an atmosphere containing 5% CO2.

### Annexin V/PI stain, CCK-8 assay and RNA interference

Oleandrin (purchased from Shanghai Yaji Biological Technology Co., Ltd) was dissolved in DMSO at an original concentration of 5ug/ul, and then diluted into proper concentration in demand. A549, H1299, HBE cells were plated in triplicate into six-well plates at proper concentration overnight before the addition of indicated treatment, then oleandrin was added. Annexin V/PI apoptosis kit (purchased from Multi Sciences Biotech Co., Ltd.) was used to detect the apoptosis rate.

CCK-8 assay kit was purchased from Bomai Meditech. The test was carried on following the manufacturer's protocol.

The XRCC1 siRNA was purchased from the GenePharma (siXRCC1: 5′-CUGGUCACCUCAUCUU UCATT-3′; siControl: 5′-UUCUCCGAACGUGUCA CGUTT-3′), transfection reagent (RNAiMax) was purchased from Invitrogen. The siRNA transfection was carried on following the manufacturer's protocol. A549 cells were transfected for 24hours, and then treated with oleandrin for 12 hours. FCM was used to detect the apoptosis rate.

### Immunofluorescence

Following indicated treatment, cells were fixed and stained. The primary antibody used was mouse monoclonal antibody against RPA (Abcam2175) and γH2AX (Abcam81299). The secondary antibodies used were Alexa Fluor 488-conjugated goat anti-mouse IgG (Molecular Probes) and Alexa Fluor 555-conjugated goat anti-mouse IgG (Molecular Probes). DNA was counterstained with DAPI. More than 300 cells were counted from random fields for each condition, and cells with more than 3 nuclear foci were calculated as positive staining.

### Cell cycle

A549 cells were planted in six-well plates, treated with oleandrin 24 hours, and cell cycle staining kit (purchased from MultiSciences) was used to detect the cell cycle following the manufacturer's protocol.

### Western blotting

Cells were lysed in RIPA buffer in the presence of 1 × protease inhibitor cocktail (Sigma). An aliquot of 50 μg total protein was run on an SDS-PAGE gel and transferred to PVDF(polyvinylidene fluoride) membrane (Millipore). This membrane was immunoblotted with mouse monoclonal antibody against XRCC1 (Abcam 1838), rabbit polyclonal antibodies against RAD51 (Santa Cruz 8349) in 5% Milk overnight. Immunoreactive proteins were visualized following the manufacturer's instructions. Densitometric analysis was performed using ImageJ 1.46 software to normalize the expression of target protein with the corresponding loading control.

### Statistical analysis

Data were presented in the form of means ± SEM, analysed with GraphPad Prism 6.0 (GraphPad software, San Diego, California, USA). Differences between groups were analysed using the One-way ANOVA (and nonparametric) (n.s. not significant; * p < 0.05; **p < 0.01; ***p < 0.001).
